# Evil Results of Over-Feeding Cattle

**Published:** 1858-09

**Authors:** 


ART. VI.—*Evil Results of Over-Feeding Cattle. A New Inquiry; fully Illustrated by Colored Engravings of the Heart, Lungs, etc. of the Diseased Prize Cattle lately exhibited by the Smithfield Cattle Club,* 1857. By FREDERICK JAMES GANT, M.R.C.S., Surgeon and Pathological Anatomist to the Royal Free Hospital. 8vo., pp. 39. London: Churchill, 1858.

ALTHOUGH the attention of the public has, during the past few years, been directed to the subject of diseased meat as occurring in the markets, yet it has not awakened that amount of interest which its importance undoubtedly demands. The London press has been especially denunciatory in its expressions in regard to the many impositions practiced upon the lower classes, and Mr. Gamgee has written several forcible letters on this subject as having a direct influence on human health. More recently, Mr. Gant has investigated the conditions of the meat of over-fed cattle as an article of food; while in our own country, the subject of hog-cholera, still-fed, tailless cows, and of swill milk, is at present the *fashionable* topic of discussion. Mr. Gant has directed his observations to the prize cattle exhibited at the "Smithfield Cattle Club," in 1857. The object of this association is to test "the best method of rearing cattle as an article of food;" and it was to see how far they had succeeded in their intentions that he visited the fair. He found that the judges had awarded the prizes to unwieldy, bulky beasts, overloaded with fat, gasping for breath, and scarcely able to move. The principal pathological changes were congestion of, and tubercles in, the lungs; the conversion of the muscular fibrillæ into fat; fatty degeneration and enlargement of the heart. In the instance of a prize steer, the heart had undergone the latter change, and it was so softened at its apex as to allow of the introduction of a blunt probe into the substance of the left ventricle, where the muscular fibres had given way. By far the most frequent variation from the normal state, was "the conversion of the heart into fat," preventing this organ from carrying on its functions to that extent necessary for the health of the animal; its contractile power was greatly diminished, causing the blood to circulate feebly, and inducing, consequently, congestion of the lungs, with enormous difficulty in breathing. Mr. Gant concludes his inquiry by suggesting that "breeders, feeders, exhibitors, and prize judges, alike visit the slaughter-houses; let them do this with a due knowledge of diseased appearances, and let them thus discover that system of rearing which is most compatible with the health of the cattle, and which produces the largest amount of nutritious food for man."

We are glad to see that the surgeon and pathologist to the Royal Free Hospital has taken this matter into consideration, and we regret that he has not pursued his inquiries further; but yet must thank him for his praiseworthy zeal in awakening attention to the subject.

Why should not our health-officers supervise our meat markets, as well as direct their attention to quarantine duties? or, why should not a competent individual, well versed in pathology, be appointed as meat inspector instead of the mere apologies for men who now hold that office?

In thus noticing this work, we do not consider that we, as physicians, are particularly concerned in rearing cattle; but we must assume that we are more than interested in its hygienic and dietetic bearings, and that medical men should supervise the rearing of cattle in regard to the nutritive qualities of food. In our own country, where agricultural fairs are so abundant, and where much attention is paid to raising stock, we may hope to look for some change in the present system.

The work is illustrated by twelve wood-cuts and seven beautifully colored engravings, and is in perfect keeping with the accustomed taste and liberality of its distinguished publisher, Mr. Churchill. We would recommend it to the attentive perusal of the scientific farmer and breeder.

